# Developmental organization of the graphic gesture with a pre-scruptural task to assess handwriting

**DOI:** 10.1192/j.eurpsy.2024.918

**Published:** 2024-08-27

**Authors:** L. Vaivre-Douret, C. Lopez

**Affiliations:** ^1^Faculty of Health, Department of Medicine, University of Paris Cité; ^2^Chair of Neurodevelopmental Clinical Phenotyping, Institut Universitaire de France (IUF), Paris; ^3^Faculty of Medicine, University of Paris-Saclay, UVSQ, INSERM Unit 1018-CESP, Villejuif; ^4^ Necker Enfants Malades University hospital, AP-HP. Centre; ^5^Department of Endocrinology, Necker-Enfants Malades hospital, IMAGINE Institute, Paris, France

## Abstract

**Introduction:**

The literature mainly focused on spatio-temporal and kinematics parameters of tracing letters or words using digitizing tablets, no recent research has previously studied the developmental prerequisites of the organization of handwriting.

**Objectives:**

We aimed to aimed to investigate and validate the developmental organization of the graphic gesture with a pre-scruptural task.

**Methods:**

122 typically developing right-handed elementary school children (grades 1^st^ to 5^th^) aged from 6 to 11;3 years old were recruited. The axe postural and arm gestural features were video-recorded with analysis in 2D reconstruction. Spatial (length, size, regularity, slope of the line…), temporal (drawing time, pause time) and kinematic measures (velocity, peak velocity) were collected with a digital pen independent connected to an analysis software tool. External validity was studied in relation with the standardized handwriting scale BHK. The child has to draw a line of cycloid loops (from left to right drawn in an anti-clockwise direction) across the width of an A4 size unlined half sheet of white paper (containing non-visible watermarks to provide the location of the pen) free to move on the table, after observing the dynamic model on the iPad placed in front of him.

**Results:**

Five main patterns of inter-segmental displacement gestures were found for the production of the line of loops with a significant developmental progress from grades 1st to 5th. Findings showed significant economic rotation movement with forearm rotation around the elbow in 4th and 5th grade, with the elbow tending significantly to be static on the table (*p* = 2.43e-16), wrist on the table and (*p* =0.02) and in half-supine position (*p* =0·001), tri-digital grasp of the pen (*p* =3.81e-08). Moreover, the mean pressure applied on the pen decrease at 4 and 5th grades and it is correlated to deleterious spatial-temporal and kinematic parameters.

**Conclusions:**

The results of our study provide the first developmental grade and age-related normative data in the developmental genesis of the graphomotor gesture and with the spatio-temporal /kinematic measures. The more mature the gesture, the more there is a decrease in degrees of freedom of movement and stabilization of the joints that is fixed, as well as the presence of a distal flexion movement extending fingers in synergy with the rotation of the arm around the elbow. Furthermore, the task of copying of loops in ecological settings appears to be a good predictor for legibility and writing speed. Those data can account for the mechanisms of motor programming necessary to the automatization of the future gesture of handwriting.

**Disclosure of Interest:**

None Declared

